# Identification and in vitro validation of prognostic lncRNA signature in head and neck squamous cell carcinoma

**DOI:** 10.1080/21655979.2021.1995577

**Published:** 2021-12-07

**Authors:** Jian Wang, Qinjiang Bian, Jialin Liu, Adili Moming

**Affiliations:** a Department of Oral and Maxillofacial Surgery, The Affiliated Stomatology Hospital of The First Affiliated Hospital of Xinjiang Medical University, Xinjiang Uyghur Autonomous Region, Urumqi, 830054, P.R. China; b Xinjiang Uygur Autonomous Region Institute of Stomatology, Xinjiang Uyghur Autonomous Region, Urumqi, 830054, P.R. China; c Department of Maxillofacial Surgery, Gansu Provincial Hospital, Lanzhou, Gansu province, 730000, P.R. China; d Department of Prosthodontics, The Affiliated Stomatology Hospital of The First Affiliated Hospital of Xinjiang Medical University, Xinjiang Uyghur Autonomous Region, Urumqi, 830054, P.R. China

**Keywords:** Head and neck squamous cell carcinoma, overall survival, lncRNAs, prognosis, TCGA

## Abstract

Long non-coding RNAs (lncRNAs) are promising cancer prognostic markers. However, the clinical significance of lncRNA signatures in evaluating overall survival (OS) outcomes of head and neck squamous cell carcinoma (HNSCC) has not been explored. This study aimed to assess the significance of lncRNA in HNSCC and to develop a lncRNA signature related to OS in HNSCC. LncRNA expression matrices were retrieved from the Cancer Genome Atlas (TCGA) data. Least Absolute Shrinkage and Selection of the Operator (LASSO), univariate and multivariate Cox regression were used for establishing a prognostic model. In vitro experiments were carried out to demonstrate the biological role of lncRNA. A prognosis model based on 7 DElncRNAs was finally established.The patients were then divided into high-risk and low-risk groups. Relative to the low-risk group, overall survival times for patients in the high-risk group were significantly low (P=2.466e−07). Risk score remained an independent prognostic factor in univariate (HR=1.329, 95%CI=1.239−1.425, p < 0.001) and multivariate (HR=1.279, 95%CI=1.184−1.382, p < 0.001) Cox regression analyses. The area under the curve (AUC) of the signature was as high as 0.78. Expressions of FOXD2-AS1  in tumor tissues were elevated, and significantly correlated with OS (P=0.008). FOXD2-AS1 silencing then significantly reduced HNSCC cell proliferation, invasion, and migration. In conclusion, a lncRNA signature was established for HNSCC prognostic prediction and FOXD2-AS1 was identified as an HNSCC oncogene.

## Introduction

Head and neck squamous cell carcinomas (HNSCC) are a cluster of cancers that occur in the mouth, oropharynx, and laryngeal regions [1]. HNSCC is the sixth most common malignancy in the world, with over 600,000 new cases diagnosed each year and 380,000 deaths worldwide [2,3]. The current treatments for HNSCC include surgery, chemotherapy, radiotherapy, and targeted therapy. Despite advancements in therapeutic technology, HNSCC is a particularly aggressive solid tumor. Local recurrence and distal metastasis are still the two major challenges of HNSCC treatments, and the current 5-year overall survival rate of HNSCC patients is about 50%, while that of advanced patients is less than 50% [4,5]. However, there is still a lack of effective clinical prognosis prediction methods for HNSCC patients [6]. Therefore, there is an urgent need to find effective markers to predict the prognosis of HNSCC, which is necessary for guiding personalized anti-cancer treatment strategies.

Long non-coding RNAs (lncRNAs), which cannot encode proteins, are RNA molecules whose transcriptional length is more than 200 nucleotides [7]. LncRNAs have been shown to regulate gene expression in a variety of ways at various levels, including epigenetic, transcriptional, post-transcriptional, translational, and post-translational, by interacting with mRNA, DNA, proteins, and miRNAs [8–10]. LncRNAs play vital roles in the pathogenesis of several cancers, including HNSCC [11]. Zheng et al found that long noncoding RNA01134 can interact with microRNA-4784 and down-regulate the expression of structure-specific recognition protein 1, thereby promoting the progression of hepatocellular carcinoma [12]. In HNSCC, Long intergenic noncoding RNA HOTAIR has been reported to be overexpressed and regulate PTEN methylation, which is involved in tumor development [13]. In addition to solid tumors, lncRNA is also considered to be related to the occurrence and development of hematological tumors. In acute myeloid leukemia, Long noncoding RNA SATB1-AS1 participates in the tolerance process of chemotherapy [14]. The expression of lncRNA varies greatly in different diseases and tissues and different disease stages. Therefore, compared with other tumor characteristics, lncRNA is a potential marker for diagnosis and prognosis [15,16]. In the past, although studies have reported that Long noncoding RNA is related to the pathogenesis and prognosis of HNSCC [6,13], there is a lack of systematic analysis of the prognosis of HNSCC and lncRNA at the omics level. In this study, we hypothesized that there is a lncRNA expression signature that is significantly related to the prognosis of HNSCC, which is promising for clinical application. We developed a lncRNA-based signature based on a large number of HNSCC patient-related data in the database and comprehensively analyzed genomic data through bio-information methods to predict the prognosis of HNSCC, and verified it in experiments *in vitro*.

## Materials and methods

### Data acquisition and processing

RNA-Seq and clinical data for HNSCC patients were retrieved from the TCGA database (https://portal.gdc.cancer.gov/). These data were extracted and integrated by downloading Perl (https://www.perl.org/get.html). RNA data were normalized using the R software (v.4.0.2 http://www.r-project.org). In total, 498 HNSCC patients were randomly assigned to the training and validation groups in a 6:4 ratio and analyzed using the R software package ‘caret.’ Differences between RNA expression patterns were analyzed using the R software ‘edgeR’ package to determine DElncRNAs. Threshold was set at | log_2_ foldchange (FC) >2.0, using adjusted P < 0.01.

### Establishment of DElncRNA-associated prognostic model

Screening of DElncRNAs with significant associations with overall survival (OS) was carried out with Univariate Cox regression analysis [17]. Validation of OS-related DElncRNAs was performed by LASSO regression using the R software ‘glmnet’ package. Then, Multivariate COX regression analyses were conducted on candidate lncRNAs obtained from LASSO regression, and a model was finally established. Risk scores for every patient were determined by the linear combination of lncRNA expression levels (x) and regression coefficient (β) from the multivariate COX regression analysis. Risk score = β_1_x_1_ + β_2_x_2_ + · ···· +β_n_x_n_.

### Confirmation of lncRNA signature

The participants were divided into high- and low-risk groups based on their median risk scores, and Kaplan Meier survival curves were drawn [18]. The univariate Cox proportional risk regression model was used to analyze data. The prognostic values were determined using an independent receiver operating characteristic (ROC) curve. Specificity, the sensitivity of risk score in predicting survival was compared to determine prognostic value. In addition, the associations between lncRNA risk score prediction and other clinical factors were verified by multivariate Cox regression. The model’s predictive accuracy was validated using the test group (n = 199).

### Cell culture and transfection

Normal Human Oral Keratinocyte (HOK) cell lines were obtained from TongPai (Shanghai) Biotechnology Co., Ltd (Shanghai, China). Human HNSCC cell lines Cal-27, FADU, and TSCCA were obtained from Procell Life Science & Technology Co., Ltd (WuHan, China). HOK cell lines were cultured in Minimum Essential Medium (MEM, HyClone, USA) with 10% fetal bovine serum (FBS) supplements (Gibco, New Zealand) and 1% penicillin plus streptomycin (BI, Israel) at 37°C in 5% CO_2_. Cal-27, FADU, and TSCCA cell lines were seeded in Dulbecco’s modified Eagle’s medium (DMEM, HyClone, USA) with 10% FBS (Gibco, New Zealand) and 1% penicillin plus streptomycin (BI, Israel). The cells were incubated at 37°C in a 5% CO_2_ atmosphere. Screening of cell lines with the highest lncRNA FOXD2-AS1 expression levels was carried out via RT-qPCR. The cells were seeded in 6-well plates, grown to 50–70% confluency, and infected with lentiviruses for lncRNA FOXD2-AS1 knockdown expression (sh-FOXD2-AS1), and knockdown expression negative control, (sh-NC) (GENECHEM, Shanghai, China). Transfection efficiency was analyzed after 72 hours.

### Extraction of total RNA and qRT-PCR analysis

The RNA extraction from cells was carried out with the Mirneasy Mini Kit extraction reagent box (QIAGEN, Germany), after which cDNA synthesis was carried out using the PrimeScript™ RT reagent Kit (TaKaRa, Japan). The qRT-PCR reaction was undertaken on SYBR® Premix Ex Taq™ (TaKaRa, Japan) using various primers: FOXD2-AS1, forward: TGGACCTAGCTGCAGCTCCA, reverse: AGTTGAAGGTGCACACACTG, and GAPDH, forward: CCCATCACCATCTTCCAGGAG, reverse: GTTGTCATGGATGACCTTGGC.

### *Fluorescence* in situ *hybridization (FISH)*

The FOXD2-AS1-FISH probe was designed and synthesized by Gemma Gene (Shanghai, China). About 1 × 10^4^ cells were inoculated overnight on a 48-well plate, fixed with 4% paraformaldehyde for 15 minutes at room temperature, aspirated the paraformaldehyde, and permeated with 0.1% Buffer. A (main component triton x 100) for 15 minutes, and incubate at 37°C for 30 minutes. Added the FISH probe to the hybridization mixture and incubated at 37°C overnight (12–18 hours). Then, the cells were washed with 20x SSC buffer. DAPI was stained to observe cell nuclei. Finally, the cells were observed under a Leica fluorescence microscope (DMi8, Leica, Germany).

### Cell proliferation analysis

Cal-27 and FADU cells (3 × 10^3^cells per well) were seeded in a 96-well plate, with 100 µl per well and 5 duplicate wells, were set. The Cell Counting Kit-8 (cck-8, APExBIO, USA) reagent (10 µl) was added to each well at 0, 24, 48, and 72 h, respectively, and incubated for 2 h. Absorbance was determined at 450 nm wavelength.

### Wound healing and Transwell migration assays

The wound healing and Transwell migration assays were carried out following the methods reported in a previously published study [19]. In short, Cal-27 and FADU cells were seeded in 6-well plates containing DMEM without FBS and scraped using 200-µl pipette tips. Cell migration and imaging were assessed at 0- and 24-hours following damage using inverted microscopy (OLYMPUSIX73, Olympus, Tokyo, Japan). Invasiveness of cells was detected using Transwell migration chamber (Corningcostar; Aperture 8 μm) coated with Matrigel (Sigma). Transfected cells were added onto the top compartment with a resuspended medium without FBS, and the bottom compartment was filled with 30% FBS. Uninvaded cells were removed from the top of the membrane after 48 hours, and invaded cells were then fixed at the bottom of the membrane and stained. Cells were counted from 5 random fields (100x magnification) and photographs were taken under a light microscope.

## Results

To identify the prognostic lncRNA signature in head and neck squamous cell carcinoma, the lncRNA expression was analyzed in HNSCC tumor and non-tumor patient samples, and specific lncRNAs correlated with overall survival outcomes of HNSCC patients were involved. Then, the prognostic lncRNA signature including 7 lncRNAs was identified by multiple regression followed by its validation in the lncRNA model. Finally, we chose FOXD2-AS1 in the 7 lncRNAs and validated the cancer-promoting function *in vitro*.

### Identification of differentially-expressed lncRNA (DElncRNAs)

**Figure 1.**
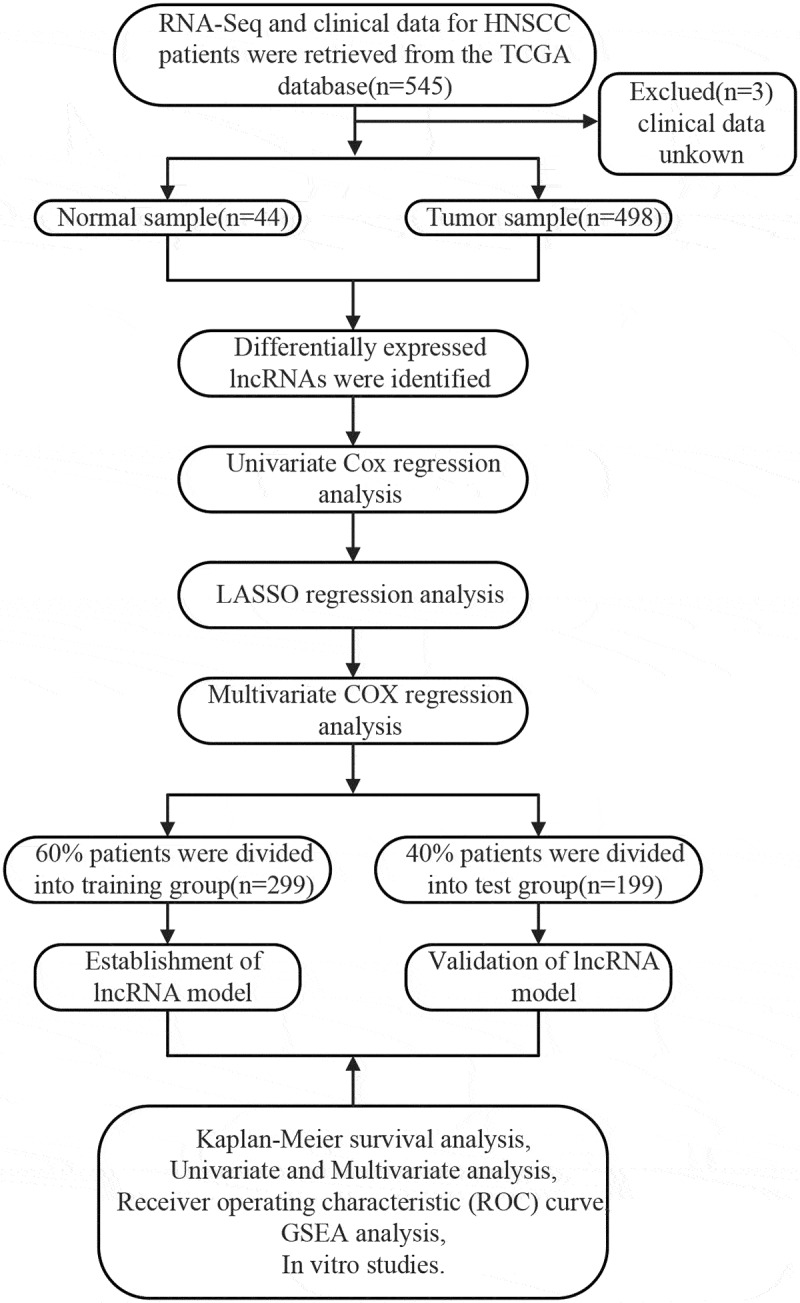
The flow chart for the methods of the bio-information analysis, which clearly showed the process of the identification of prognostic lncRNA signature in HNSCC

Figure 1 depicts the flow chart of the biological information analysis approach used in this study. A total of 1,023 DElncRNAs (735 upregulated and 288 downregulated) were identified in tumor and non-tumor samples using the R Project ‘edger’ package. A volcano plot **(Figure S1A)** and heat maps **(Figure S1B)** of DElncRNAs were constructed.

### Establishment of the lncRNA signature

**Figure 2.**
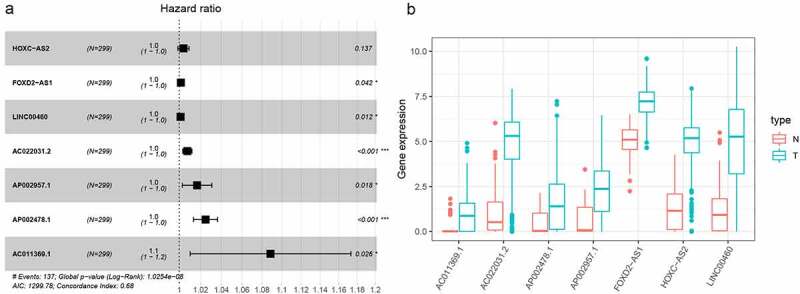
LASSO regression analysis was used to select DElncRNAS related to prognosis of HNSCC. (a) Forest plots showing the relationship between different lncRNA subsets and OS in the training queue. Unadjusted HRs are 95% CIs. (b) The box diagram shows the differential gene expression of model lncRNA in TCGA database. ***P < 0.001, **P < 0.01, and *P < 0.05

In this study, 72 DElncRNAs were found to have a significant correlation with overall survival outcomes (P < 0.01) **(Supplementary Table 1)**. DElncRNAs were further verified by Lasso regression and 10 DElncRNAs were obtained for the model to achieve the best performance **(Figure S2A, B)**. The 10 DElncRNAs were then analyzed using multiple regression. Findings showed that 7 HNSCC prognosis-related DElncRNAs including HOXC-AS2, FOXD2-AS1, LINC00460, AC022031.2, AP002957.1, AP002478.1, and AC011369.1 were obtained. The Forest plot for the relationship between each lncRNA and OS is shown in Figure 2a. Each lncRNA coefficient is shown in **Supplementary Tables 2**. The expression of lncRNAs was considerably upregulated in HNSCC, according to TCGA transcriptome data (Figure 2b).

### Validation of the lncRNA signature

**Figure 3.**
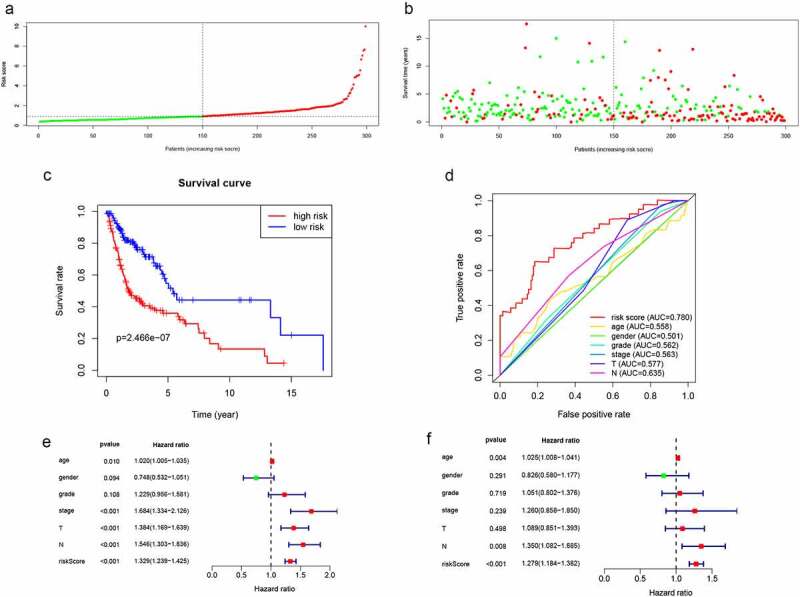
Validation of lncRNA signaling in predicting the prognosis of HNSCC in the training group. (a) Risk score distribution of patients in the training group. (b) Survival status distribution of high- and low-risk patients. (c) Kaplan-Meier OS curves were constructed for low- and high-risk HNSCC patients. (d) Comparison of the predictive OS ability of the risk score model with other clinical parameters using ROC curve analysis for HNSCC patients. (e) Relationship between risk factors and HNSCC OS (univariate Cox regression analysis). (f) Relationship between risk factors and HNSCC OS (multivariate Cox regression analysis)

The risk scores for each patient were computed using the expression levels and risk coefficients of seven prognostic lncRNAs in the model. Patients’ allocation into low- and high-risk groups was done using the median risk score. Expression levels of 7 lncRNAs were distributed along with the training group as shown in **Figure S3**. The risk scores and the survival time distribution for the training group are shown in Figure 3a and b, respectively. Kaplan-Meier curves of low- as well as high-risk groups, are shown in Figure 3c. The high-risk patients were found to have shorter survival times (P = 2.466e−07) compared with low-risk patients. In addition, the prognostic model based on lncRNA biomarkers had an AUC of 0.78, which was higher than other indicators including age, gender, and tumor stage (Figure 3d). Furthermore, univariate analysis revealed that the risk score had a significant association with OS (HR = 1.329, 95%CI = 1.239 − 1.425, P < 0.001) (Figure 3e). In the multivariate analysis, the risk score was shown to be an independent prognostic factor in HNSCC (HR = 1.279, 95%CI = 1.184 − 1.382, P< 0.001) (figure 3f). Therefore, the lncRNA signature had better HNSCC prognostic abilities, when compared with the other indicators.

### Validation and verification of the lncRNA model

**Figure 4.**
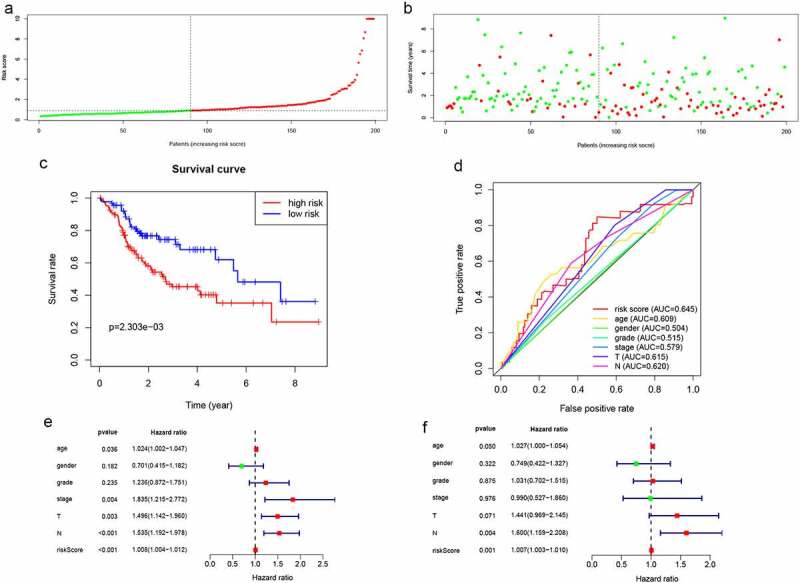
Validation of lncRNA signaling in predicting the prognosis of HNSCC in the validation group. (a-d) The results of lncRNA signature prediction of HNSCC outcomes in the validation group were consistent with the results of the training cohort(Figure 3). (e) Relationship between risk factors and HNSCC OS in the validation group (univariate Cox regression analysis). (f) Relationship between risk factors and HNSCC OS in the validation group (multivariate Cox regression analysis)

Distributions of lncRNA expressions, risk scores, as well as survival times in the validation group are shown in **Figure S4**and Figure 4a-b. Kaplan-Meier curves of low- and high-risk groups are shown in Figure 4c. In the validation group, OS for low-risk patients was significantly high, when compared to the high-risk patients (P = 2.303e−03). The AUC of the validation group was 0.645 (Figure 4d). The lncRNA model was an independently associated prognostic factor in the validation cohort (Figure 4e,f).

### Expression levels of FOXD2-AS1 in HNSCC tissues are significantly elevated and correlated with poor prognostic outcomes

**Figure 5.**
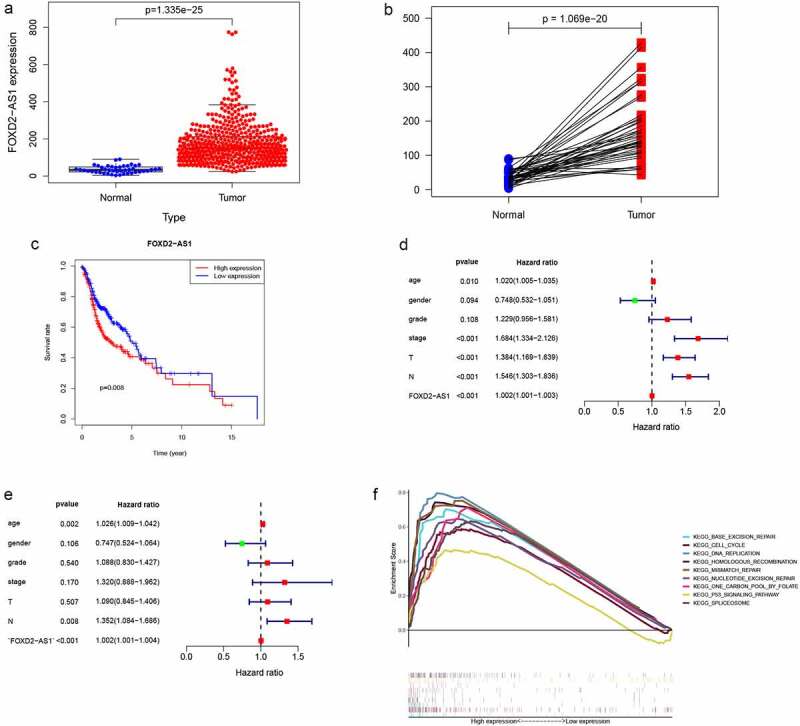
Levels of FOXD2-AS1 is significantly increased in HNSCC tissues and associated with poor prognosis. (a) The expression of FOXD2-AS1 in HNSCC tissues (n = 501) compared with normal tissues (n = 44) was analyzed using TCGA data. (b) FOXD2-AS1 level was verified in 44 paired HNSCC carcinoma and paracarcinoma tissues from the TCGA database. (c) Kaplan-meier plotter was used to determine the relationship between FOXD2-AS1 levels and overall survival in HNSCC patients. (d-e) Univariate and multivariate COX regression analysis showed that FOXD2-AS1 level was an independent predictor of HNSCC. (f) GSEA describes the biological pathways associated with FOXD2-AS1

The expression of the LncRNA FOXD2-AS1 is abnormal in a range of tumors, and it is associated with patient survival and prognosis [13]. Therefore, the role of FOXD2-AS1 in HNCSS needs to be investigated further. The current study evaluated the transcription level of FOXD2-AS1 in HNSCC by evaluating the TCGARNA-seq data. In tumor tissues, FOXD2-AS1 expressions were significantly elevated, compared to normal tissues (Figure 5a). In addition, we analyzed FOXD2-AS1 expressions in tumors and adjacent normal tissues by TCGA data (Figure 5b). Patients with low levels of FOXD2-AS1 expression had considerably longer survival times than those with elevated levels of expression (Figure 5c). Furthermore, FOXD2-AS1 was found to be an independent predictor in HNSCC patients (Figure 5d,e).

### FOXD2-AS1 functional analysis and its relationship with HNSCC clinicopathological characteristics

**Figure 6.**
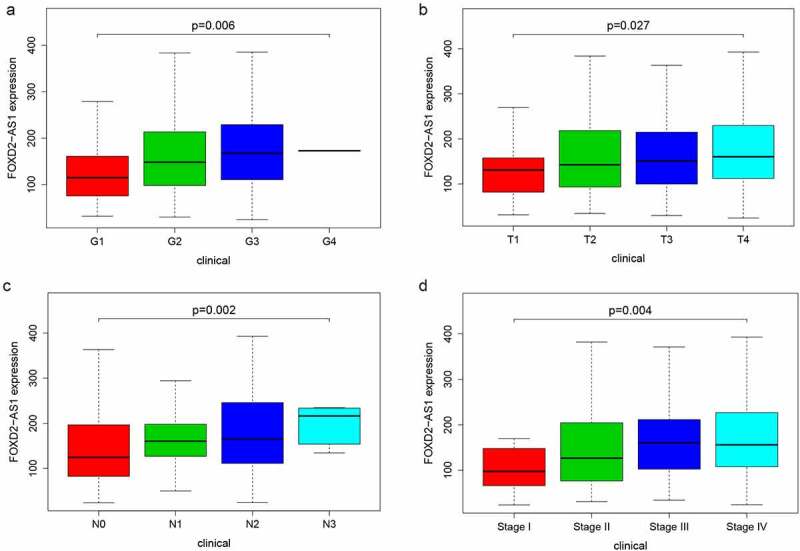
Clinicopathologic features of FOXD2-AS1 expression. (a-d) FOXD2-AS1 expression significantly increased in advanced cases

GSEA analysis was undertaken in the current study to determine the biological effects of FOXD2-AS1. High scores of FOXD2-AS1 were shown to be significantly enriched in pathways such as cell cycle, base excision repair, DNA replication, and the p53 signaling pathway (figure 5f). The relationship between lncRNA expression levels and clinico-pathological features was determined using TCGA data. Expressions of FOXD2-AS1 were significantly correlated with grade (P = 0.006), T stage (P = 0.027), N stage (P = 0.002), and TNM stage (P = 0.004) (Figure 6a-d).

### FOXD2-AS1 was amplified in HNSCC, and suppressing FOXD2-AS1 inhibited cell proliferation and migration in HNSCC cells

**Figure 7.**
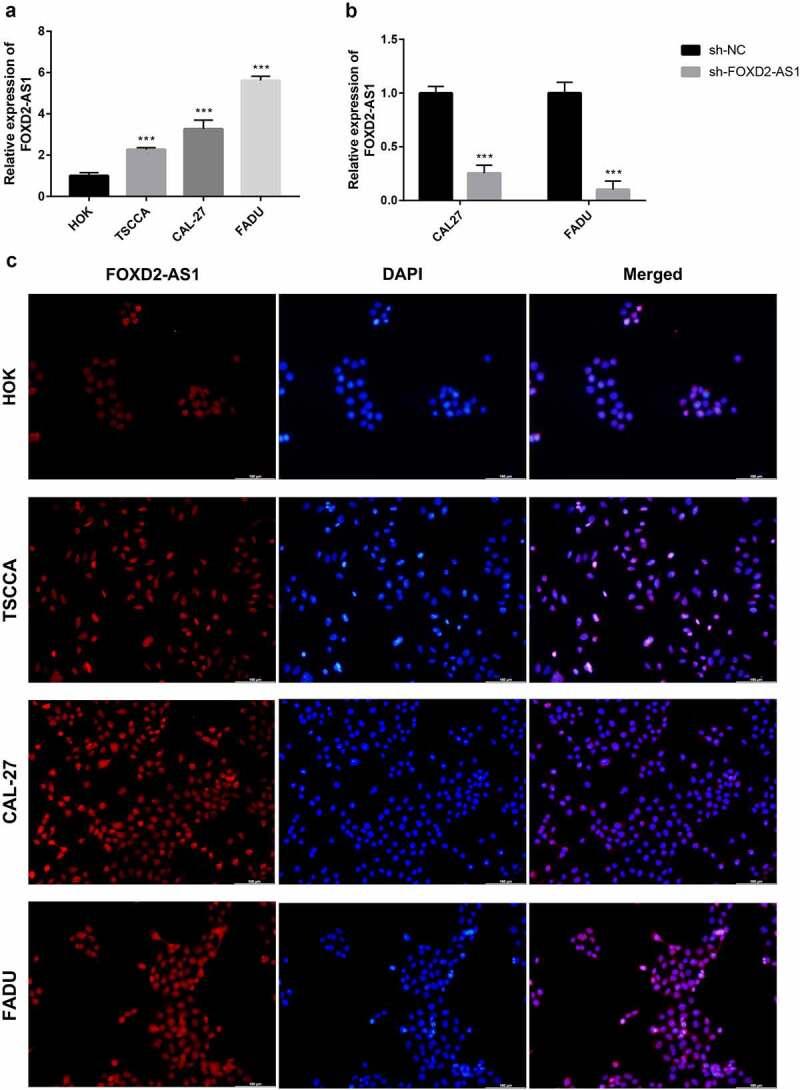
The expression and biological characteristics of FOXD2-AS1 in HNSCC in vitro study. (a) FOXD2-AS1 was remarkably increased in HNSCC cell lines. (b) Transfection efficiency was verified after lentivirus transfection of FOXD2-AS1 or negative control shRNA. (c) Expression and localization of FOXD2-AS1 in HOK, TSCCA, CAL-27 and FADU cell lines

**Figure 8.**
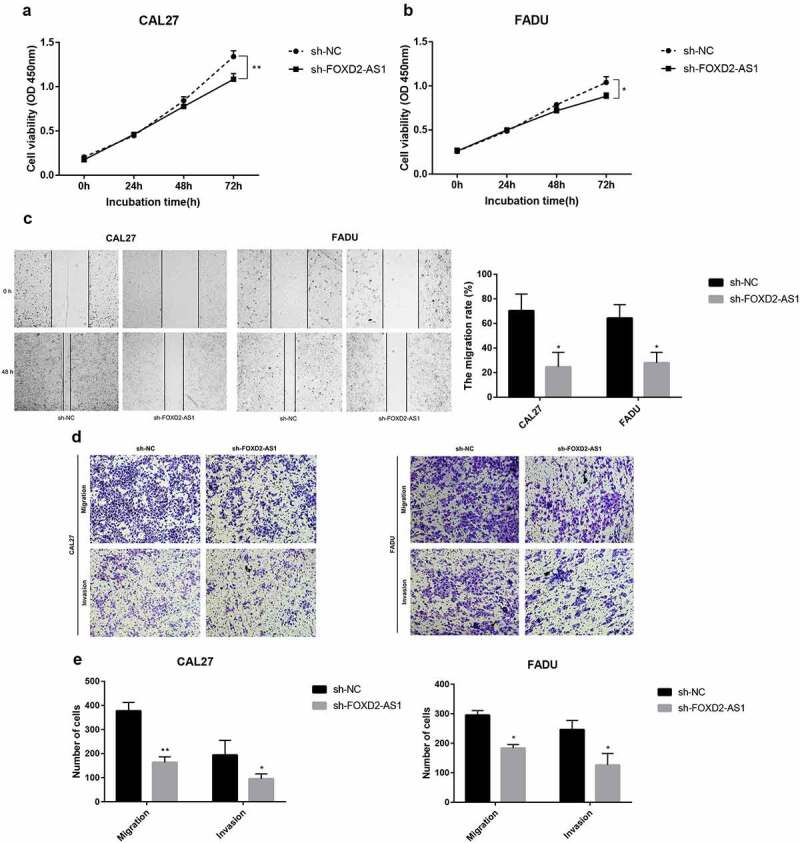
The expression and biological characteristics of FOXD2-AS1 in HNSCC in vitro study. (a-b) The viability of HNSCC cells was assessed by CCK-8 assay at 0, 24, 48 and 72 h after transfection. (c) Images were recorded 0 and 48 hours after the cell scratch experiment. (d-e) Transwell assay was used to detect the invasion and migration of HNSCC cells. ***P < 0.001, **P < 0.01, and *P < 0.05

FOXD2-AS1 expression levels were detected in HNSCC cell lines (CAL27, FADU, and TSCCA) and normal HOK cell lines. In the HNSCC cell lines, FOXD2-AS1 was highly expressed when compared to the normal HOK cell lines, whereas expression levels of CAL27 and FADU were higher compared with that of TSCCA. CAL27 and FADU cell lines were, therefore, selected for further experiments (Figure 7a). The current study evaluated cell transfection efficiency using qRT-PCR and established that FOXD2-AS1 expression levels were significantly reduced after shRNA transfection (Figure 7b). Meanwhile, the expression and localization of FOXD2-AS1 have been verified by FISH experiments in cell lines including HOK, TSCCA, CAL-27, FADU cell lines. The results showed that FOXD2-AS1 expression levels were higher in HNSCC cell lines (TSCCA, CAL-27, FADU) compared with normal HOK cell lines. Moreover, FOXD2-AS1 was mainly located in the nucleus of these cell lines, as shown in Figure 7c. CCK-8 was used to establish the effects of FOXD2-AS1 knockdown on cell proliferation. When FOXD2-AS1 was silenced, CAL27 and FADU cell proliferation was significantly reduced compared to sh-NC cell proliferation (Figure 8a,b). Furthermore, wound healing tests showed that FOXD2-AS1 silencing significantly inhibited wound healing in the two cell lines (Figure 8c). The invasion and migration rates of FADU cells and CAL27 transfected with shRNA were significantly suppressed as compared with those transfected with sh-NC (Figure 8d,e).

## Discussion

According to previous studies, HNSCC patients are prone to recurrence and metastasis, indicating the urgent need for aggressive research into novel biomarkers and biomarker-based therapies to improve survival [20]. In recent years, lncRNAs have received a lot of attention as emerging regulators with a variety of biological functions [21]. lncRNAs are transcripts with over 200 nucleotides and are involved in several cellular properties, including cell growth, pluripotency, and cell differentiation [22]. Several studies have suggested that dysregulated lncRNAs could influence cancer progression [23,24]. Many lncRNAs are potential biomarkers and targets for cancer diagnosis and treatment [25]. Several prognostic models based on lncRNA have been developed for tumors [17,18,26]. Previous studies have also reported the association between Long noncoding RNA and the pathogenesis and prognosis of HNSCC [6,13], but there is a lack of systematic analysis of Long noncoding RNA at the omics level in HNSCC. The current study used bioinformatics and univariate Cox analysis to evaluate OS-related DElncRNAs using HNSCC transcriptome data from the TCGA database. A total of 72 OS-related DElncRNAs were identified. Lasso regression analysis was used to modify the list of candidate DElncRNAs. Multivariate Cox regression analysis showed prognostic signatures including 7 DElncRNAs. Kaplan-Meier, Cox regression analyses as well as time-dependent ROC curves were then used to verify the prognostic value of the lncRNA signature, which were considered independent predictors of HNSCC prognosis. Further validation in the internal validation set was undertaken in the current study.

Previous studies have reported that FOXD2-AS1 is associated with tumorigenesis and development. According to studies, FOXD2-AS1 is up-regulated in a variety of cancers, including gastric cancer, bladder cancer, nasopharyngeal cancer, lung cancer, esophageal cancer, liver cancer, thyroid cancer, colorectal cancer, and skin cancer, and promotes cancer cell proliferation, migration, and invasion, and is negatively correlated with prognosis [27]. The mechanism by which FOXD2-AS1 promotes tumor progression has been reported differently in different tumors. FOXD2-AS1 can regulate microRNA; Ni et al. [28] reported that overexpression of FOXD2-AS1 promotes glioma growth via modulating the miR-185-5P/HMGA2 axis and PI3K/AKT signaling pathways. In rectal cancer, up-regulated FOXD2-AS1 has been reported to regulate miR-25-3p/Sema4c Axis to promote the invasion and metastasis of colorectal cancer cells [29]; in addition, FOXD2-AS1 is abnormally elevated in breast cancer tissues and promotes FOXD2‑AS1/miR‑150‑5p/PFN2 axis, accelerating the growth of transplanted tumors *in vivo* [30]. Conversely, knock-down of FOXD2-AS1 can inhibit tumor progression. Knockdown of FOXD2-AS1 can inhibit the progression of gallbladder cancer by regulating the methylation of MLH1 [31]. FOXD2-AS1 is highly expressed in proliferative hemangioma tissues, and its knockdown inhibits cell proliferation, migration, invasion, and colony formation through the miR-324-3p/PDRG1 pathway [32]. FOXD2-AS1 depletion can also regulate the expression of CDX1 and inhibit the proliferation of cervical cancer cells [33]. Therefore, FOXD2-AS1 induces cancer-promoting activities, which are mostly thought to be due to its interaction with microRNAs, thereby altering gene expression or epigenetic modification. The mechanism may be different in different cancers. But the mechanism also may be similar, and FOXD2-AS1 changes the entire gene expression network. In this study, a single-gene analysis of FOXD2-AS1 was undertaken to evaluate the role of FOXD2-AS1 in HNSCC. We compared FOXD2-AS1 expression levels in the tumor and normal or adjacent tissues in the TCGA-HNSCC cohort. Both unpaired and paired tests indicated increased expression levels of FOXD2-AS1 in HNSCC. FOXD2-AS1 expression levels were significantly correlated with clinicopathological characteristics, including grade, T stage, TNM stage, and N stage. Patients with elevated FOXD2-AS1 expression levels had worse prognostic outcomes compared with those with low FOXD2-AS1 expression levels. We found that FOXD2-AS1 independently predicted tumor prognosis. Furthermore, GSEA analysis of FOXD2-AS1 expression data in TCGA-HNSCC was performed. The findings revealed that FOXD2-AS1 influences the occurrence and progression of HNSCC through 9 pathways. Some of these biological effects of related pathways on tumors have been reported in the literature, such as cell cycle regulation. Several post-translational modifications control cell cycle progression, and cyclin-dependent kinases (CDKs) are key cell cycle regulators [34]. Zhang et al. [35] reported that lncRNA CASC11 enhanced gastric cancer cell proliferation, migration as well as invasion by regulating the expression of cell cycle-related protein CDK1. Studies by Qin et al. [36] have shown that microRNA-99a-5p inhibits breast cancer progression as well as cell cycle pathway by down-regulating cell cycle-related protein CDC25A. Furthermore, inhibiting CDK4/6 increases the radiosensitivity of HPV-negative HNSCC [37]. In addition, the role of FOXD2-AS1 in HNSCC was also related to the p53 signaling pathway, and the p53 signaling pathway plays a part in the regulation of expression of several genes and is closely related to the development of human tumors, whose activation initiates cell cycle arrest as well as DNA repair, and inhibits proliferation of OSCC cells [38]. Overexpression of hsa-miR-128a-mediated p53 signaling pathway increases the role of Pembrolizumab in Laryngeal Squamous Cell Carcinoma Cell lines [39]. The findings of the current study confirmed that FOXD2-AS1 is elevated in HNSCC tissues and is a potential predictor of HNSCC in patients, thereby promoting the proliferation of HNSCC cell lines. The current study’s findings also revealed that FOXD2-AS1 has an impact on HNSCC biology via these pathways. However, this potential mechanism should be explored in future research.

There were certain limitations to the current study, which have been noted. Firstly, the study lacked a significant number of clinical specimens to detect differential expression of lncRNAs, and only a database was used for paired analysis. Second, no further *in vivo* or *in vitro* research has been done to confirm the prognostic value of the proposed lncRNA signatures. Instead, bioinformatics was used to infer from online data sets. Finally, lncRNAs lacked regulatory mechanisms. The current study intends to refine these findings in the future.

## Conclusion

In conclusion, we identified novel lncRNA signatures including HOXC-AS2, FOXD2-AS1, LINC00460, AC022031.2, AP002957.1, AP002478.1, and AC011369.1 that could predict HNSCC prognosis independently, and we furtherly validated the cancer-promoting function of FOXD2-AS1 in HNSCC *in vitro*, which was similar to that in other kinds of cancers. Our findings may contribute to the creation of prognostic prediction systems for HNSCC in the clinic. However, the current study’s findings need to be validated, and the functions of another six lncRNAs in HNSCC advancement have yet to be investigated.

## Supplementary Material

Supplemental Material

## Data Availability

This study obtained open data from The Cancer Genome Atlas (TCGA) database (https://portal.gdc.cancer.gov/).

## Highlights

- Identification of DElncRNAs in tumor and non-tumor samples using the R ‘edger’ package.
- Analysis of lncRNA expression in HNSCC tumor and non-tumor patient samples.
- Establishment of lncRNA signature for prognostic prediction of HNSCC.
- Identification of FOXD2-AS1 as an oncogene for HNSCC.

## References

[1] Economopoulou P, De Bree R, Kotsantis I, et al. Diagnostic tumor markers in Head and Neck Squamous Cell Carcinoma (HNSCC) in the clinical setting. Front Oncol. 2019;9:827. 31555588 10.3389/fonc.2019.00827 PMC6727245

[2] Miyauchi S, Kim SS, Pang J, et al. Immune modulation of head and neck squamous cell carcinoma and the tumor microenvironment by conventional therapeutics. Clin Cancer Res. 2019;25(14):4211–4223. 30814108 10.1158/1078-0432.CCR-18-0871 PMC6635035

[3] Wang X, Cao K, Guo E, et al. Identification of immune-Related LncRNA pairs for predicting prognosis and immunotherapeutic response in head and neck squamous cell carcinoma. Front Immunol. 2021;12:658631. 33995377 10.3389/fimmu.2021.658631 PMC8116744

[4] Wang HC, Chan LP, Cho SF. Targeting the immune microenvironment in the treatment of head and neck squamous cell carcinoma. Front Oncol. 2019;9:1084. 31681613 10.3389/fonc.2019.01084 PMC6803444

[5] Ebnoether E, Diagnostic ML. Therapeutic applications of exosomes in cancer with a special focus on Head and Neck Squamous Cell Carcinoma (HNSCC). Int J Mol Sci. 2020;21(12):4344. 10.3390/ijms21124344 PMC7352611 32570802

[6] Yang QQ, Deng YF. (2014) Long non-coding RNAs as novel biomarkers and therapeutic targets in head and neck cancers. Int J Clin Exp Pathol. 2014;7(4):1286–1292. 24817925 PMC4014209

[7] Xing C, Sun SG, Yue ZQ, et al. Role of lncRNA LUCAT1 in cancer. Biomed Pharmacother. 2021;134:111158. 33360049 10.1016/j.biopha.2020.111158

[8] Ulitsky I, Bartel DP. lincRNAs: genomics, evolution, and mechanisms. Cell. 2013;154(1):26–46. 23827673 10.1016/j.cell.2013.06.020 PMC3924787

[9] Fatica A, Bozzoni I. Long non-coding RNAs: new players in cell differentiation and development. Nat Rev Genet. 2014;15(1):7–21. 24296535 10.1038/nrg3606

[10] Zhang X, Wang W, Zhu W, et al. Mechanisms and functions of long non-coding RNAs at multiple regulatory levels. Int J Mol Sci. 2019;20(22):5573. 10.3390/ijms20225573 PMC6888083 31717266

[11] Zhao J, Liu D, Yang H, et al. Long noncoding RNAs in head and neck squamous cell carcinoma: biological functions and mechanisms. Mol Biol Rep. 2020;47(10):8075–8090. 32914266 10.1007/s11033-020-05777-w

[12] Zheng S, Guo Y, Dai L, et al. Long intergenic noncoding RNA01134 accelerates hepatocellular carcinoma progression by sponging microRNA-4784 and downregulating structure specific recognition protein 1. Bioengineered. 2020;11(1):1016–1026. 32970959 10.1080/21655979.2020.1818508 PMC8291876

[13] Li D, Feng J, Wu T, et al. Long intergenic noncoding RNA HOTAIR is overexpressed and regulates PTEN methylation in laryngeal squamous cell carcinoma. Am J Pathol. 2013;182(1):64–70. 23141928 10.1016/j.ajpath.2012.08.042

[14] Zhou H, Jia X, Yang F, et al. Long noncoding RNA SATB1-AS1 contributes to the chemotherapy resistance through the microRNA-580/ 2ʹ-5ʹ-oligoadenylate synthetase 2 axis in acute myeloid leukemia. Bioengineered. 2021;12(1):6403–6417. 34516354 10.1080/21655979.2021.1971508 PMC8806783

[15] Yan X, Hu Z, Feng Y, et al. Comprehensive genomic characterization of long non-coding RNAs across human cancers. Cancer Cell. 2015;28(4):529–540. 26461095 10.1016/j.ccell.2015.09.006 PMC4777353

[16] Li W, Chen QF, Huang T, et al. Identification and validation of a prognostic lncrna signature for hepatocellular carcinoma. Front Oncol. 2020;10:780. 32587825 10.3389/fonc.2020.00780 PMC7298074

[17] Xia P, Li Q, Wu G, et al. An immune-related lncRNA signature to predict survival in glioma patients. Cell Mol Neurobiol. 2021;41(2):365–375. 32410107 10.1007/s10571-020-00857-8 PMC11448555

[18] Ma B, Li Y, Ren Y. Identification of a 6-lncRNA prognostic signature based on microarray re-annotation in gastric cancer. Cancer Med. 2020;9(1):335–349. 31743579 10.1002/cam4.2621 PMC6943089

[19] Pijuan J, Barceló C, Moreno DF, et al. In vitro cell migration, invasion, and adhesion assays: from cell imaging to data analysis. Front Cell Dev Biol. 2019;14(7):107. 10.3389/fcell.2019.00107 PMC6587234 31259172

[20] Muzaffar J, Bari S, Kirtane K, et al. Recent advances and future directions in clinical management of head and neck squamous cell carcinoma. Cancers (Basel). 2021;13(2):338. 33477635 10.3390/cancers13020338 PMC7831487

[21] Li Z, Yan M, Yu Y, et al. LncRNA H19 promotes the committed differentiation of stem cells from apical papilla via miR-141/SPAG9 pathway. Cell Death Dis. 2019;10(2):130. 30755596 10.1038/s41419-019-1337-3 PMC6372621

[22] Liang Y, Song X, Li Y, et al. A novel long non-coding RNA-PRLB acts as a tumor promoter through regulating miR-4766-5p/SIRT1 axis in breast cancer. Cell Death Dis. 2018;9(5):563. 29752439 10.1038/s41419-018-0582-1 PMC5948209

[23] Zhuo W, Liu Y, Li S, et al. Long noncoding RNA GMAN, Up-regulated in gastric cancer tissues, is associated with metastasis in patients and promotes translation of ephrin A1 by competitively binding GMAN-AS. Gastroenterology. 2019;156(3):676–691. 30445010 10.1053/j.gastro.2018.10.054

[24] Hu Q, Ye Y, Chan LC, et al. Oncogenic lncRNA downregulates cancer cell antigen presentation and intrinsic tumor suppression. Nat Immunol. 2019;20(7):835–851. 31160797 10.1038/s41590-019-0400-7 PMC6619502

[25] Chi Y, Wang D, Wang J, et al. Long non-coding RNA in the pathogenesis of cancers. Cells. 2019;8(9):1015. 10.3390/cells8091015 PMC6770362 31480503

[26] Li W, Chen QF, Huang T, et al. Identification and validation of a prognostic lncRNA signature for hepatocellular carcinoma. Front Oncol. 2020;10:780. 32587825 10.3389/fonc.2020.00780 PMC7298074

[27] Hu Q, Tai S, Wang J. Oncogenicity of lncRNA FOXD2-AS1 and its molecular mechanisms in human cancers. Pathol Res Pract. 2019;215(5):843–848. 30723052 10.1016/j.prp.2019.01.033

[28] Ni W, Xia Y, Bi Y, et al. FoxD2-AS1 promotes glioma progression by regulating miR-185-5P/HMGA2 axis and PI3K/AKT signaling pathway. Aging (Albany NY). 2019;11(5):1427–1439. 30860979 10.18632/aging.101843 PMC6428107

[29] Zhang M, Jiang X, Jiang S, et al. LncRNA FOXD2-AS1 regulates miR-25-3p/Sema4c axis to promote the invasion and migration of colorectal cancer cells. Cancer Manag Res. 2019;11:10633–10639. 31908535 10.2147/CMAR.S228628 PMC6927494

[30] Jiang M, Qiu N, Xia H, et al. Long non‑coding RNA FOXD2‑AS1/miR‑150‑5p/PFN2 axis regulates breast cancer malignancy and tumorigenesis. Int J Oncol. 2019;54(3):1043–1052. 30628646 10.3892/ijo.2019.4671

[31] Gao J, Dai C, Yu X, et al. Silencing of long non-coding RNA FOXD2-AS1 inhibits the progression of gallbladder cancer by mediating methylation of MLH1. Gene Ther. 2021;28(6):306–318. 32917950 10.1038/s41434-020-00187-w

[32] Zhao T, Zhang J, Ye C, et al. lncRNA FOXD2-AS1 promotes hemangioma progression through the miR-324-3p/PDRG1 pathway. Cancer Cell Int. 2020;20(1):189. 32489325 10.1186/s12935-020-01277-w PMC7247140

[33] Chen DZ, Wang TF, Dai WC, et al. LncRNA FOXD2-AS1 accelerates the progression of cervical cancer via downregulating CDX1. Eur Rev Med Pharmacol Sci. 2019;23(23):10234–10240. 31841177 10.26355/eurrev_201912_19660

[34] Xiao Y, Lucas B, Molcho E, et al. Inhibition of CDK1 activity by sumoylation. Biochem Biophys Res Commun. 2016;478(2):919–923. 27520372 10.1016/j.bbrc.2016.08.051 PMC5002384

[35] Zhang L, Kang W, Lu X, et al. LncRNA CASC11 promoted gastric cancer cell proliferation, migration and invasion in vitro by regulating cell cycle pathway. Cell Cycle (Georgetown, Tex). 2018;17(15):1886–1900. 10.1080/15384101.2018.1502574 PMC6152531 30200804

[36] Qin H, Liu W. MicroRNA-99a-5p suppresses breast cancer progression and cell-cycle pathway through downregulating CDC25A. J Cell Physiol. 2019;234(4):3526–3537. 30443946 10.1002/jcp.26906

[37] Göttgens EL, Bussink J, Leszczynska KB, et al. Inhibition of CDK4/CDK6 enhances radiosensitivity of HPV negative head and neck squamous cell carcinomas. Int J Radiat Oncol Biol Phys. 2019;105(3):548–558. 31271827 10.1016/j.ijrobp.2019.06.2531

[38] Bidaud P, Chasle J, Sichel F, et al. Expression of p53 family members and CD44 in oral squamous cell carcinoma (OSCC) in relation to tumorigenesis. Histol Histopathol. 2010;25(3):331–339. 20054805 10.14670/HH-25.331

[39] Chen H, Guo Y, Huang J, et al. Upregulating hsa-miR-128a increased the effects of pembrolizumab on laryngeal cancer cells via the p53 pathway. Biomed Res Int. 2021;2021:2342784. 33791361 10.1155/2021/2342784 PMC7997759

