# Supplementary material for: Identification and in vitro validation of prognostic lncRNA signature in head and neck squamous cell carcinoma
Source: Bioengineered. 2021 Dec 7;12(2):10049–62. doi: 10.1080/21655979.2021.1995577 (PMC8809959; doi:10.1080/21655979.2021.1995577)
Supplement: Supplemental Material [file KBIE_A_1995577_SM8452.zip › Supplementary Figures legends.docx]

Sup Figures:

1. The differentially expressed lncRNAS (DElncRNAS) in TCGA-HNSCC. (A) Heatmaps of differentially expressed lncRNAS for HNSCC and non-tumor (control) tissues. (B) Volcano plot shows the differentially expressed lncRNAS. Red and green indicate up- and down -regulation, respectively.
2. LASSO regression analysis was used to select DElncRNAS related to prognosis of HNSCC. (A) In univariate Cox regression analysis, LASSO coefficient profiles of some important DElncRNAS were obtained. (B) Optimal parameter selection of DElncRNA in LASSO Model (λ) of HNSCC.
3. Heat map of associated lncRNA expression profiles in the prognostic signature in training group.
4. Heat map of associated lncRNA expression profiles in the prognostic signature in validation group..
