# Supplementary figures and images for: Identification and in vitro validation of prognostic lncRNA signature in head and neck squamous cell carcinoma
Source: Bioengineered. 2021 Dec 7;12(2):10049–62. doi: 10.1080/21655979.2021.1995577 (PMC8809959; doi:10.1080/21655979.2021.1995577)

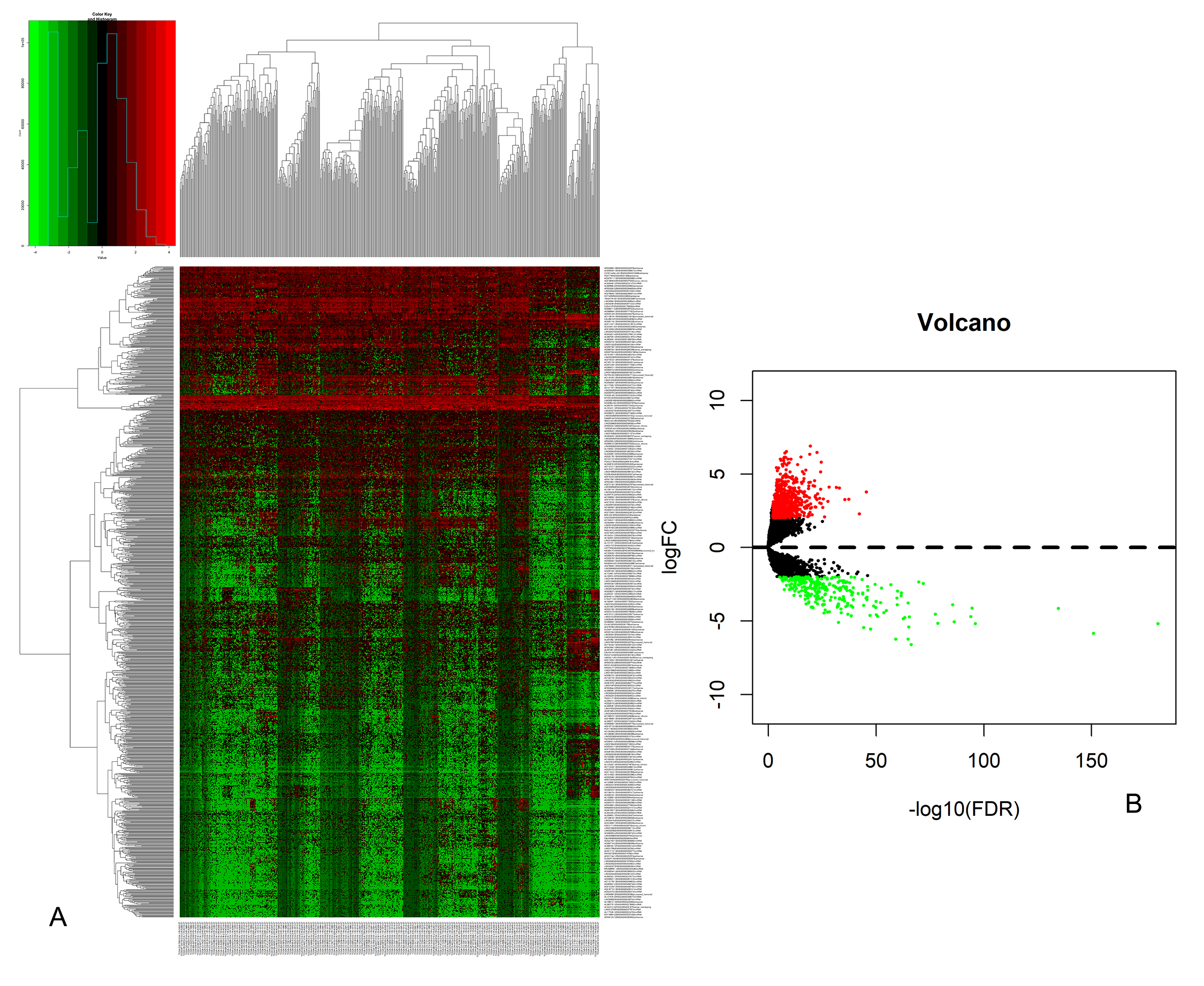

Supplement: Supplemental Material [file KBIE_A_1995577_SM8452.zip › supplement 1.tif]

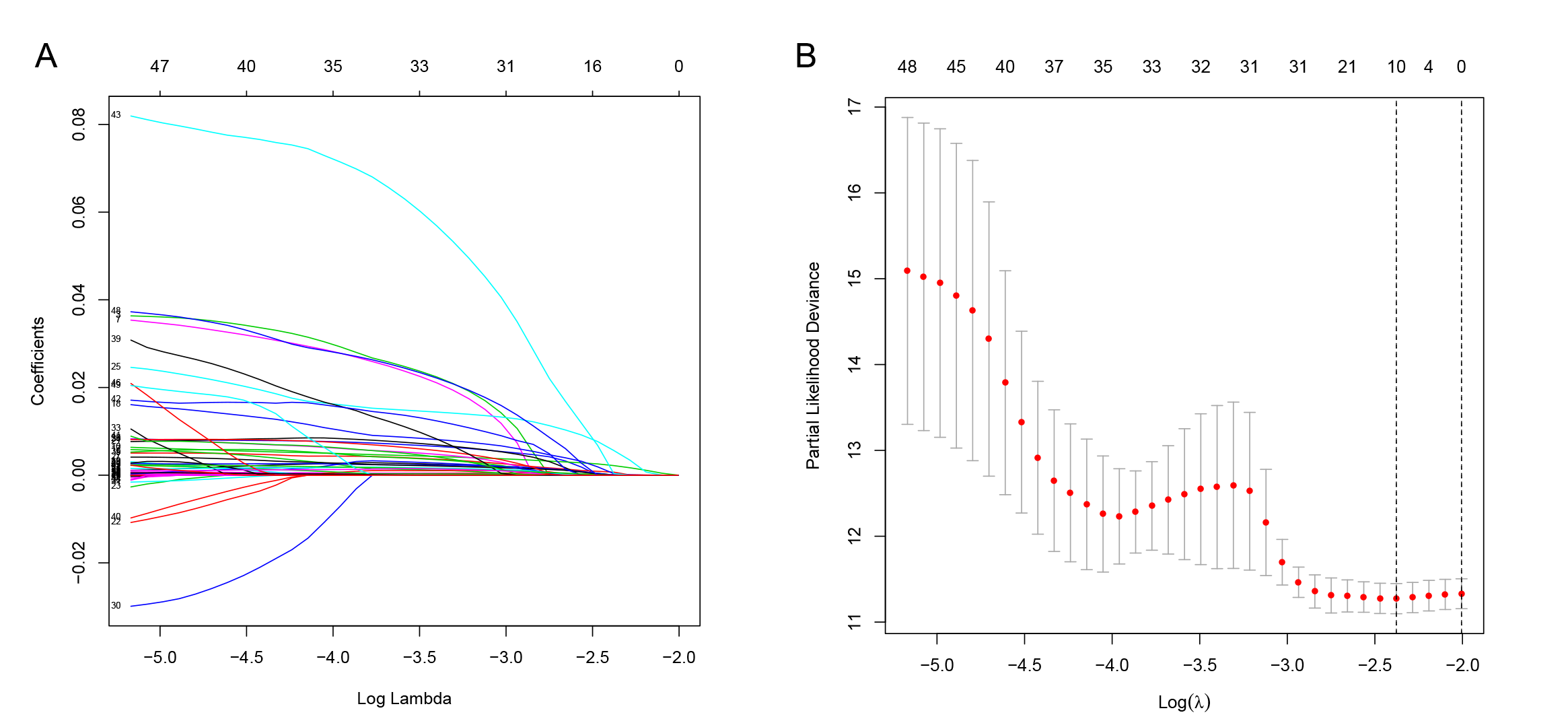

Supplement: Supplemental Material [file KBIE_A_1995577_SM8452.zip › supplement 2.tif]

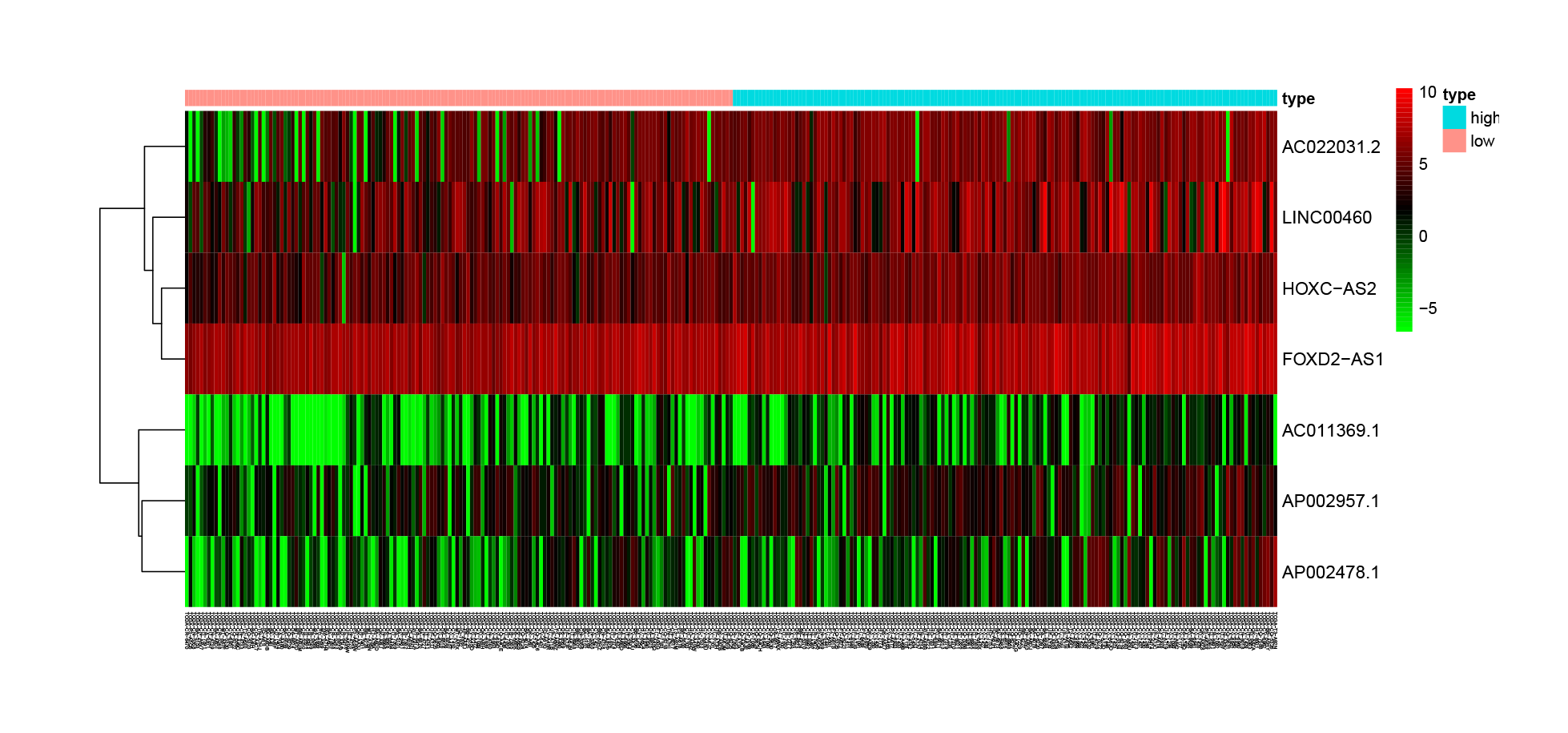

Supplement: Supplemental Material [file KBIE_A_1995577_SM8452.zip › supplement 3.tif]

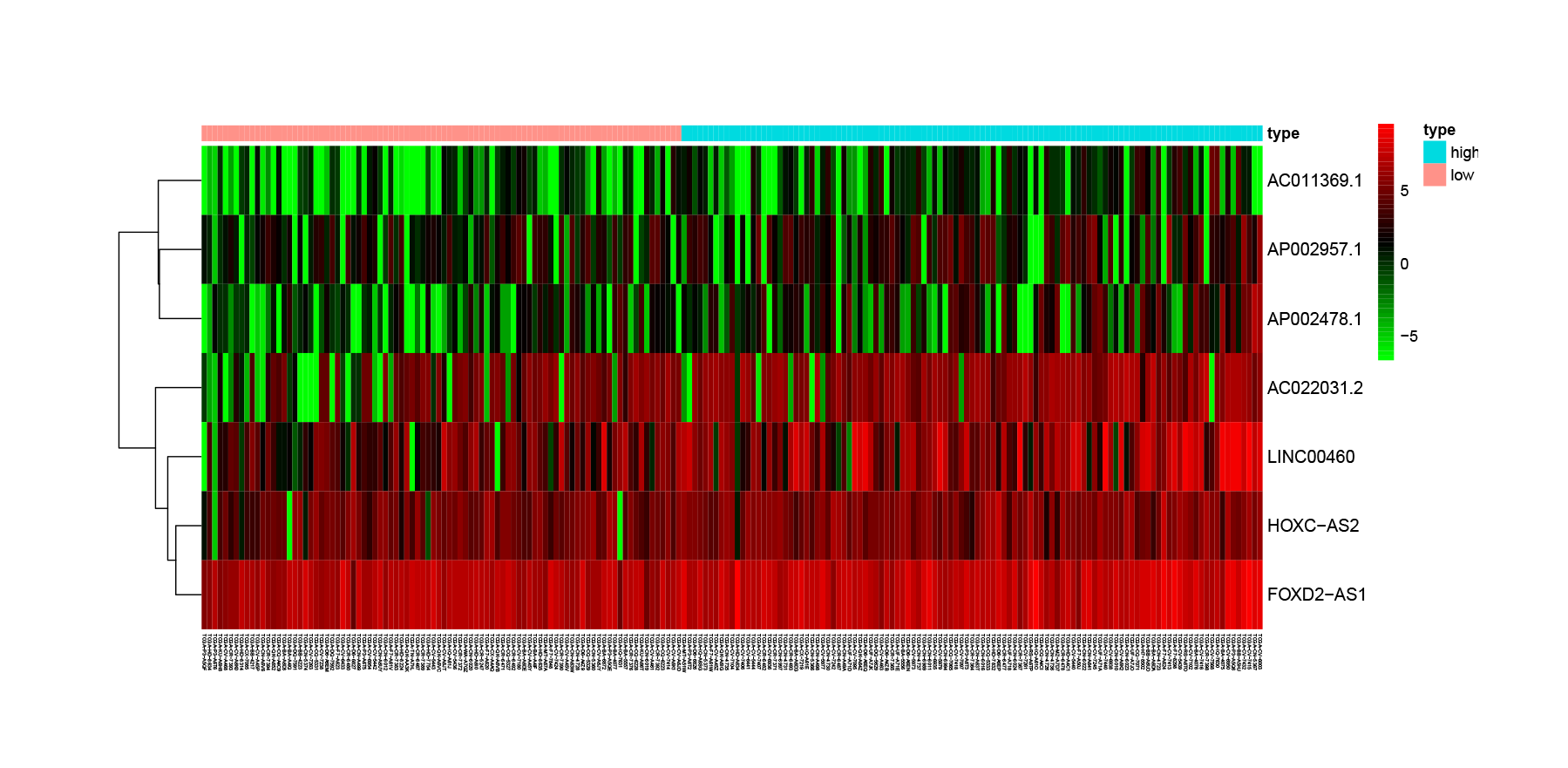

Supplement: Supplemental Material [file KBIE_A_1995577_SM8452.zip › supplement 4.tif]
